# Transcutaneous tibial nerve electrostimulation in the treatment of neurogenic overactive bladder syndrome after ischemic stroke

**DOI:** 10.61622/rbgo/2026rbgo43

**Published:** 2026-05-29

**Authors:** Marcia Maria Gimenez, Rodrigo Aquino Castro, Rebecca Sotelo Pinheiro da Silva, Maria Augusta Tezelli Bortolini, Fatima Fani Fitz, Marcia Maiumi Fukujima, Emerson Oliveira, Marair Gracio Ferreira Sartori

**Affiliations:** 1 Universidade Federal de São Paulo São Paulo SP Brazil Universidade Federal de São Paulo, São Paulo, SP, Brazil.; 2 Centro Universitário FMABC Santo André SP Brazil Centro Universitário FMABC, Santo André, SP, Brazil.

**Keywords:** Ischemic stroke, Urinary incontinence, Transcutaneous electrical nerve stimulation, Overactive bladder, Neurogenic bladder

## Abstract

**Objective:**

To prospectively evaluate the effects of transcutaneous tibial nerve electrical stimulation (TTNS) on urinary symptoms and quality of life in women with neurogenic overactive bladder (nOAB) secondary to ischemic stroke (IS).

**Methods:**

This was a prospective case series with a six-month follow-up. Women with clinically and radiologically confirmed IS who developed nOAB symptoms persisting for more than six months after the cerebrovascular screened for eligibility. TTNS was administered twice weekly for six weeks, with 30 minute-minute sessions. Electrical stimulation was delivered at the sensory threshold using a fixed frequency of 10Hz and a pulse width of 250 µs. Outcomes were evaluated at baseline, immediately after treatment, and at six months using a one-week bladder diary and the King's Health Questionnaire (KHQ). Statistical analysis was performed using the Friedman test for repeated measures, with a significance threshold of p < 0.05.

**Results:**

Among 236 screened women with IS, 34 (21%) reported persistent nOAB symptoms; 31 completed the study protocol and were included in the final analysis. Mean age was 59.3 ± 13.6 years, and 67.7% were postmenopausal. Detrusor overactivity was identified in 51.6% of urodynamic studies. TTNS produced significant reductions in daytime frequency (11.95 to 6.99 voids/day post-treatment; 7.21 at follow-up), urgency episodes (13.32 to 0.97; 1.35 at follow-up), urge incontinence (9.19 to 0.26; 0.58 at follow-up), and nocturia (2.99 to 0.40; 0.64 at follow-up) (all p < 0.001). Significant improvements were observed across all KHQ domains, including general health perception (58.06 ± 24.48 to 30.65 ± 22.09), incontinence impact (60.22 ± 24.97 to 11.83 ± 22.02), physical and social limitations, emotional well-being, sleep and energy, and severity measures (all p < 0.001). Improvements were sustained at six months, with partial symptom recurrence in 19.6% and complete recurrence in 6.7% of participants.

**Conclusion:**

TTNS was associated with significant and sustained improvements in urinary symptoms and quality of life in women with post-stroke nOAB.

## Introduction

Neurogenic overactive bladder (nOAB) is characterized by urinary urgency, with or without urge incontinence, frequently accompanied by increased daytime frequency and nocturia, occurring in individuals with neurological disorders and at least partially preserved bladder sensation.^(1)^ In these conditions, OAB symptoms arise from impaired central control of micturition, and are commonly observed in multiple sclerosis (MS), spinal cord injury (SCI), Parkinson's disease (PD), and stroke.^(2)^ The severity and clinical expression of urinary symptoms reflect the type, location, and extent of neurological impairment.^(3)^ The prevalence of neurogenic OAB in the general adult population is estimated at approximately 0.6%, according to community-based epidemiological data from the Boston Area Community Health (BACH) study.^(4)^ Among specific neurological conditions, urinary incontinence attributable to neurogenic detrusor overactivity is reported in approximately 50% of patients with MS and 52% of those with SCI, while lower rates are observed in PD (33%) and stroke populations (24%).^(5,6)^ These data underscore the substantial burden of bladder dysfunction among individuals with neurological disease, particularly after ischemic stroke (IS).

IS is the most common cerebrovascular event and is defined as an episode of neurological dysfunction caused by focal cerebral, spinal, or retinal infarction, confirmed by neuroimaging evidence of ischemic injury in a defined vascular territory.^(7)^ IS may disrupt suprapontine inhibitory pathways involved in micturition control – particularly within the anteromedial frontal lobe, paraventricular white matter, and putamen – leading to detrusor overactivity and impaired voluntary voiding control.^(3)^ In previous work from our group, we demonstrated a significant association between ischemic stroke topography and urinary incontinence in women, with frontal lobe involvement predominating among incontinent patients, reinforcing the critical role of suprapontine structures in continence mechanisms.^(8)^ Post-stroke urinary symptoms may persist beyond motor recovery and are associated with poorer rehabilitation outcomes, prolonged hospitalization, and reduced quality of life.^(9,10)^ Early recognition and appropriate management of bladder dysfunction are therefore essential to optimize recovery and prevent long-term complications.^(11)^ Pharmacological management of nOAB relies primarily on antimuscarinic agents and β_3_-adrenergic agonists, although tolerability frequently limits long-term adherence. Antimuscarinics are associated with adverse effects such as dry mouth, constipation, and cognitive impairment—particularly concerning in post-stroke patients—while mirabegron, although generally better tolerated, may increase blood pressure and heart rate.^(3,11,12)^ Intradetrusor botulinum toxin injections can provide temporary symptom relief but are limited by high costs, the need for repeated procedures, and the risk of urinary retention requiring intermittent catheterization.^(11,12)^

Given these limitations, transcutaneous tibial nerve stimulation (TTNS) has emerged as a promising, noninvasive neuromodulation strategy for nOAB. Derived from principles originally described in traditional Chinese acupuncture near the Sanyinjiao (SP6) region, TTNS involves electrical stimulation of the tibial nerve—a mixed motor and sensory nerve arising from the L4–S3 roots, which also contribute to bladder and pelvic floor innervation. Activation of somatic afferent fibers modulates sacral spinal reflexes and central micturition pathways, thereby, reducing detrusor overactivity.^(13)^

As a low-cost outpatient therapy, TTNS has demonstrated reductions in urinary frequency, urgency, nocturia, and incontinence episodes in patients with PD and MS, with favorable tolerability compared with oral anticholinergics.^(13-15)^ Reported adverse events are generally mild and transient, most commonly local discomfort at the stimulation site, and contraindications are limited, mainly including pacemakers and local infection at the electrode site.^(16)^ However, despite increasing evidence in other neurological disorders, data on TTNS specifically for nOAB secondary to IS remain scarce. Given the substantial burden of post-stroke nOAB and the limitations of current treatments, TTNS may represent a safe, accessible, and effective therapeutic option. Accordingly, this study aimed to evaluate the effects of TTNS on urinary symptoms and quality of life in women with nOAB secondary to IS.

## Methods

This prospective case series was conducted between 2016 and 2018 in the Emergency Medicine Sector and the Urogynecology and Vaginal Surgery Outpatient Clinic of the Department of Gynecology at Universidade Federal de São Paulo (UNIFESP) - Escola Paulista de Medicina (EPM). Women with nOAB secondary to clinically and radiologically confirmed IS were eligible if urinary symptoms had persisted for more than six months. This report adheres to the key elements of the CARE (CAse REport) guidelines for case series^(17)^ and includes a detailed description of the intervention based on the TIDieR (Template for Intervention Description and Replication) checklist.^(18)^

Women with clinically and radiologically confirmed IS were included. IS was defined as an acute focal neurological deficit consistent with cerebral infarction^(7)^ and was diagnosed by a neurologist based on clinical evaluation, with confirmation by neuroimaging (computed tomography or magnetic resonance imaging). Participants who developed nOAB symptoms persisting for more than six months after the event were included. Eligible participants presented with urgency, frequency, nocturia, or urge urinary incontinence consistent with nOAB, without evidence of local pathological or metabolic causes. Urodynamic studies were performed in all participants to document bladder function patterns, including detrusor overactivity; however, urodynamic confirmation was not required as an inclusion criterion.

Exclusion criteria included cognitive impairment (assessed using the Mini-Mental State Examination [MMSE]),^19^ presence of a cardiac pacemaker, pregnancy, poorly controlled diabetes, untreated urinary tract infection, or preexisting urinary dysfunction. MMSE cutoff scores were adjusted for educational level: 13 for illiterate participants, 18 for those with up to 4 years of schooling, 21 for those with 4–6 years, and 26 for those with higher education.

Patients completed the Portuguese version of the King's Health Questionnaire (KHQ), a validated instrument assessing the impact of urinary symptoms on quality of life.^(20)^ The questionnaire was administered at three time points: baseline, immediately after treatment, and six months post-treatment. To enhance the clinical interpretability of quality-of-life outcomes, changes in KHQ domain scores were analyzed according to the minimal clinically important difference (MCID), defined as the smallest change perceived as meaningful by patients. Based on previously published anchor- and distribution-based approaches, a reduction of at least 5 points in each KHQ domain was considered clinically relevant, while reductions ≥10 points were interpreted as moderate clinical improvement.^(21)^

In parallel, participants completed a one-week bladder diary at the same time points, recording urinary frequency, urgency episodes, urge incontinence, and nocturia to provide objective symptom tracking and support treatment outcome assessment.

The transcutaneous tibial nerve stimulation (TTNS) protocol was performed twice weekly for six weeks (12 sessions in total) using a Quark Dualpex 961 device (Quark Medical, São Paulo, Brazil).

Each session was conducted with the patient seated comfortably, and surface electrodes were applied using conductive gel and adhesive tape. One electrode was placed posterior to the medial malleolus (retromalleolar), and the second electrode was positioned approximately 10 cm proximally along the medial aspect of the lower leg. Proper placement was confirmed when the patient reported tingling sensations radiating to the sole and toes, indicating stimulation of the tibial nerve distribution.

Stimulation parameters consisted of biphasic, rectangular, charge-balanced pulses delivered at a frequency of 10 Hz and a pulse width of 250 µs. The intensity was individually adjusted to remain below the motor threshold, ensuring adequate sensory stimulation without discomfort. Each session lasted 30 minutes and was administered by a trained physiotherapist experienced in neuromodulation therapy. In cases of unilateral neurological deficit or local skin irritation, electrode placement was alternated between limbs to maintain patient comfort and treatment consistency.

The same physiotherapist conducted all KHQ assessments and supervised every TTNS session, ensuring strict adherence to the study protocol and continuous monitoring for adverse effects. At each evaluation point, bladder diaries and KHQ questionnaires were reviewed in detail, and any discrepancies were clarified directly with participants. Treatment adherence was tracked through attendance logs documenting session participation.

Because this study was designed as an exploratory prospective case series, no a priori sample size calculation was undertaken. To provide quantitative context for the final sample, a post hoc sample size assessment was performed based on the established minimal clinically important difference (MCID) of 10 points for the Incontinence Impact domain of the King's Health Questionnaire (KHQ). Calculations assumed a two-sided α of 0.05 and were based on the observed within-subject variability of paired measurements to estimate the statistical sensitivity of the sample.

Statistical analyses were performed using SPSS version 11.5 (IBM Corp., Armonk, NY, USA), Minitab 14, and Microsoft Excel XP. Descriptive statistics were expressed as means, medians, and standard deviations. Nonparametric tests were applied, including the Friedman and Wilcoxon signed-rank tests for longitudinal comparisons. Confidence intervals were calculated when appropriate, and statistical significance was defined as p < 0.05.

The study protocol was reviewed and approved by the institutional Ethics Committee (CEP 1540/04), and written informed consent was obtained from all participants before inclusion.

## Results

**Figure 1:**
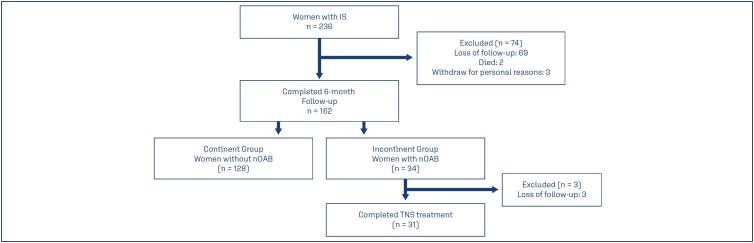
Flow diagram of participant recruitment, eligibility, inclusion, and follow-up

A total of 236 women diagnosed with IS were initially screened for eligibility. Of these, 162 completed the six-month clinical follow-up, and 34 (21%) reported symptoms consistent with nOAB. Among these 34 women, three were excluded due to loss to follow-up, resulting in 31 participants who completed the study protocol and were included in the final analysis (Figure 1).

Participants were predominantly middle-aged (mean age 59.3 ± 13.6 years), postmenopausal (67.7%), married (54.8%), and self-identified as White (67.7%), with a mean BMI of 29.8 ± 7.9 kg/m². Obstetric history revealed a multiparous profile (mean 6.10 ± 4.58 pregnancies), with predominance of vaginal deliveries (mean 5.16 ± 4.56), while cesarean sections (mean 0.45 ± 1.03) and miscarriages (mean 0. 55 ± 1.09) were less frequent. The mean time since stroke was 9 ± 2 months. At the index event, stroke severity according to the NIHSS was classified as minor (0–4) in 22 women (71%), moderate (5–15) in 8 (25.8%), and moderate-to-severe (>15) in 1 (3.2%). Right-hemisphere lesions predominated (61.3%). All participants presented with hemiparesis (100%). Regarding ambulatory status, 27 women (87.1%) were ambulatory without aid and 4 (12.9%) required assistance; none were non-ambulatory. Functional disability according to the modified Rankin Scale (mRS) showed no or no significant disability (0–1) in 25 women (80.6%), slight disability (2) in 2 (6.5%), and moderate disability (3) in 4 (12.9%), with no cases ≥4. The mean Barthel Index score was 95 ± 8.5, and the mean MMSE score was 26.3 ± 2.4. Urodynamic findings demonstrated detrusor overactivity in 15 participants (48.4%), urodynamic stress incontinence in 4 (12.9%), mixed urodynamic findings (DO + USI) in 1 (3.2%), and normal urodynamic findings in 11 (35.5%). When considering the presence of any DO component (isolated or associated), detrusor overactivity was identified in 16 participants, corresponding to 51.6% of the cohort. A comprehensive summary of baseline clinical, obstetric, urodynamic, and neurological characteristics is presented in table 1.

**Table 1:** Baseline clinical, obstetric, urodynamic, and neuroanatomical characteristics of the study population

Characteristic	n(%)	Mean ± SD	Median	CV(%)	95%CI
Age (years)	-	59.35 ± 13.61	57	22.9	4.79
					
Menopausal status					
Premenopausal	10(33.3)	-	-	-	-
Postmenopausal	21(67.7)	-	-	-	-
					
Ethnicity					
White	21(67.7)	-	-	-	-
Black or mixed race	10(32.3)	-	-	-	-
					
Marital status					
Married	17(54.8)	-	-	-	-
Separated	3(9.7)	-	-	-	-
Widowed	8(25.8)	-	-	-	-
					
Body mass index (kg/m²)	-	29.81 ± 7.90	28	26.5	2.78
					
Obstetric history					
Pregnancies (n)		6.10 ± 4.58	4	75.1	1.61
Vaginal deliveries (n)	-	5.16 ± 4.56	4	88.4	1.61
Cesarean deliveries (n)	-	0.45 ± 1.03	0	227.5	0.36
Miscarriages (n)		0.55 ± 1.09	0	198.9	0.38
					
Cerebral lesion laterality					
Right hemisphere	19(61.3)	-	-	-	-
Left hemisphere	12(38.7)	-	-	-	-
					
Urodynamic findings					
Detrusor overactivity	16(51.6)	-	-	-	-

Values are expressed as mean ± standard deviation (SD), median, coefficient of variation (CV), and 95% confidence interval (CI) where applicable. Percentages refer to the proportion of the total study population (n=31)

The one-week bladder diaries demonstrated statistically significant reductions in urinary frequency, urgency, urge incontinence and nocturia across the three evaluation points: baseline, post-treatment and six-month follow-up (Table 2).

**Table 2:** Bladder diary parameters at baseline, post-treatment, and six-month follow-up

Parameter	Mean	Median	SD	CV(%)	95%CI	p-value
Frequency (voids/day)						
Baseline	11.95					
Post-treatment	6.99	7	1.90	27.1	0.67	<0.001*
Follow-up	7.21	7	2.32	32.2	0.82	
Urinary urgency (episodes/day)						
Baseline	13.32	13	8.22	61.7	2.89	
Post-treatment	0.97	0	1.89	195	0.66	<0.001*
Follow-up	1.35	0	2.82	208	0.99	
Urge incontinence (episodes/day)						
Baseline	9.19	9	8.23	89.5	2.90	
Post-treatment	0.26	0	1.00	387	0.35	<0.001*
Follow-up	0.58	0	1.78	307	0.63	
Nocturia (voids/night)						
Baseline	2.99	3	1.95	65.1	0.69	
Post-treatment	0.40	0	0.57	143	0.20	<0.001*
Follow-up	0.64	0	1.07	168	0.38	

CV = coefficient of variation; CI = confidence interval;

* p-values refer to comparisons across time points (Friedman test, significance threshold p < 0.05)

Analysis of the KHQ demonstrated significant post-treatment improvements across all evaluated domains (Table 3). Significant reductions were observed in scores related to general health perception, incontinence impact, limitations in daily, physical and social activities, emotional well-being, sleep and energy, and symptom severity (all p < 0.001). Improvements were sustained at six months, with minimal symptom recurrence -partial in six women (19.6%) and complete in two (6.7%) indicating maintenance of treatment response over time.

**Table 3:** King's Health Questionnaire domain scores at baseline, post-treatment, and six-month follow-up

Parameter		Mean ± SD	Median	CV(%)	95%CI	p-value
General Health						
	Baseline	58.06 ± 24.48	75	42.2	8.62	
	Post-treatment	30.65 ± 22.09	25	72.1	7.78	<0.001*
	Follow-up	30.65 ± 24.76	25	80.8	8.71	
Incontinence Impact						
	Baseline	60.22 ± 24.97	67	41.5	8.79	
	Post-treatment	11.83 ± 22.02	0	186.2	7.75	<0.001*
	Follow-up	15.05 ± 27.00	0	179.3	9.50	
Daily Activity Limitations						
	Baseline	46.24 ± 27.12	50	58.6	9.55	
	Post-treatment	6.99 ± 13.45	0	192.5	4.74	<0.001*
	Follow-up	12.90 ± 20.95	0	162.4	7.38	
Physical Limitations						
	Baseline	62.90 ± 24.99	67	39.7	8.80	
	Post-treatment	10.22 ± 22.64	0	221.6	7.97	<0.001*
	Follow-up	15.59 ± 28.20	0	180.9	9.93	
Social Limitations						
	Baseline	38.71 ± 24.66	33	63.7	8.68	
	Post-treatment	6.45 ± 14.56	0	225.8	5.13	<0.001*
	Follow-up	9.32 ± 19.10	0	213.5	7.00	
Personal Relationships						
	Baseline	45.24 ± 29.88	33	66.1	12.78	
	Post-treatment	6.06 ± 13.16	0	217.1	5.50	<0.001*
	Follow-up	11.59 ± 23.27	0	200.7	9.51	
Emotions						
	Baseline	48.39 ± 30.97	44	64.0	10.90	
	Post-treatment	9.68 ± 20.64	0	213.2	7.26	<0.001*
	Follow-up	11.11 ± 20.89	0	188.0	7.35	
Sleep and energy						
	Baseline	54.84 ± 27.62	67	50.4	9.72	
	Post-treatment	8.06 ± 20.13	0	249.6	7.08	<0.001*
	Follow-up	11.83 ± 22.44	0	189.7	7.90	
Severity Measures						
	Baseline	48.12 ± 22.02	50	45.8	7.75	
	Post-treatment	7.80 ± 17.99	0	230.8	6.33	<0.001*
	Follow-up	9.41 ± 18.10	0	192.3	6.37	

Values are expressed as mean ± standard deviation (SD), median, coefficient of variation (CV), and 95% confidence interval (CI) where applicable. *p* values refer to comparisons across time points (Friedman test. significance threshold *p* < 0.05).

Adverse events were monitored throughout the intervention period. No serious adverse events or treatment discontinuations related to TTNS were recorded.

## Discussion

This prospective case series demonstrated that transcutaneous tibial nerve stimulation (TTNS) was associated with significant and sustained improvements in lower urinary tract symptoms in women with neurogenic overactive bladder secondary to ischemic stroke. After six weeks of treatment, daytime urinary frequency decreased from 11.95 to 6.99 voids/day, urgency from 13.32 to 0.97 episodes/day, urge urinary incontinence from 9.19 to 0.26 episodes/day, and nocturia from 2.99 to 0.40 voids/night (all p < 0.001). These improvements were largely maintained at six months, with partial recurrence in 19.6% and complete recurrence in 6.7% of participants. Improvements were also observed across all domains of the King's Health Questionnaire (KHQ), with mean reductions ranging from 27.4 points (General Health Perception) to more than 50 points (Physical Limitations), exceeding established minimal clinically important difference (MCID) thresholds (≥5–10 points).

To contextualize statistical sensitivity, a post hoc power analysis was performed using the MCID of 10 points for the Incontinence Impact domain of the KHQ. Based on the observed within-subject variability and a two-sided α of 0.05, approximately 45 participants were required to achieve 80% power to detect a 10-point difference. With the final sample of 31 women, the estimated power to detect the MCID was approximately 63–65%. However, the observed mean reduction of 48.39 points suggests a large effect size, reducing the likelihood of a type II error in the primary outcome.

These findings are consistent with the physiological basis of tibial nerve stimulation. Amarenco et al.^(22)^ demonstrated acute inhibitory urodynamic effects of posterior tibial nerve stimulation supporting its mechanistic rationale. Subsequent studies in neurological populations, including multiple sclerosis^(13)^ and Parkinson's disease^(14,15)^ have reported improvements in urgency, frequency, nocturia and quality of life following tibial nerve stimulation. In post-stroke populations, Monteiro et al.^(23)^ reported sustained improvements in urinary symptoms for up to 12 months in men with nOAB, suggesting durability of neuromodulatory effects.

Cândido et al.^(24)^ conducted a randomized controlled trial comparing tibial nerve electrostimulation and parasacral electrostimulation in women with post-stroke nOAB. Both groups received 12 sessions (twice weekly), and outcomes were assessed immediately after treatment. Significant improvements in urinary frequency, urgency, and incontinence episodes were observed in both groups, with no statistically significant difference between therapies. Quality of life, measured by the ICIQ-SF and WHOQOL-BREF, also improved after treatment. However, no medium-term follow-up was performed, and the bladder diary was limited to three days. In contrast, the present study used a one-week bladder diary and the KHQ, a condition-specific instrument for urinary incontinence, and included six-month follow-up. Thus, while Cândido et al.^(24)^ provide comparative evidence, the current study adds information regarding magnitude and sustainability of symptom reduction.

Similarly, Silva et al.^(25)^ reported a case series of four women with post-stroke neurogenic bladder treated with 12 sessions of posterior tibial nerve electrical stimulation. Their protocol used a pulse width of 700μs, whereas the present study employed biphasic pulses of 250μs at 10 Hz, adjusted below motor threshold. Silva et al. observed reductions in urinary frequency and improvements in selected KHQ domains; however, no statistically significant differences were detected, likely due to the small sample size (n = 4). In contrast, the present study included 31 women and demonstrated statistically significant reductions in all bladder diary parameters and across all KHQ domains (p < 0.001), with changes exceeding MCID thresholds and sustained at six months. Some individuals may require retreatment to maintain long-term efficacy.

Van der Pal et al.^(26)^ evaluated maintenance protocols following initial TTNS therapy and reported progressive deterioration in continence, nocturia, and voided volume after discontinuation. In the present study, partial recurrence in approximately one-fifth of participants suggests that periodic maintenance sessions may be necessary in selected cases.

Although TTNS is minimally invasive and well tolerated, comparisons across studies should be interpreted cautiously. Differences in patients’ characteristics, stimulation parameters, outcome measures, and concomitant therapies may influence results. In this study, TTNS was evaluated as an isolated intervention. Despite the observed improvements, limitations should be acknowledged. The single-arm design limits causal inference and does not allow exclusion of placebo effects or natural symptom variation. The sample size restricted subgroup analyses, and the six-month follow-up does not permit conclusions regarding long-term durability. Participant self-selection may also have introduced bias.

Nevertheless, the prospective design, standardized protocol, and use of validated instruments strengthen the internal consistency of the findings. This study addresses a clinically vulnerable and underrepresented population—women with post-stroke neurogenic overactive bladder—for whom pharmacological or invasive therapies may be limited. The results support the need for adequately powered randomized controlled trials to confirm efficacy and define long-term management strategies.

## Conclusion

This prospective case series suggests that TTNS is a safe, feasible, and well-tolerated therapeutic option for women with nOAB secondary to IS – a clinically vulnerable and underrepresented population with limited treatment alternatives. TTNS was associated with significant and sustained improvements in urinary symptoms and health-related quality of life over six months. As a noninvasive and low-cost, neuromodulation strategy, TTNS may broaden therapeutic options for post-stroke bladder dysfunction while avoiding the systemic adverse effects associated with pharmacological therapy and the procedural risks of invasive interventions.

## Data Availability

The research data are described in the article presented.
